# Socioeconomic inequalities on unmet needs for mental health care: a cross-section analysis in European Union countries

**DOI:** 10.1192/j.eurpsy.2025.307

**Published:** 2025-08-26

**Authors:** J. V. Santos, V. Pinheiro

**Affiliations:** 1 University of Porto; 2 RISE-Health / CINTESIS; 3 ULS Santo António; 4 University of Porto / ULS Matosinhos, Porto, Portugal

## Abstract

**Introduction:**

Mental health disorders are a leading cause of disability in European Union countries. Previous evidence highlighted the role of socioeconomic inequalities on unmet mental health care needs, varying by income or education. Being both reducing inequalities’ gaps and mental health promotion current goals of European Union (EU), it is essential to understand the differences between EU countries and the role of socioeconomic factors on this.

**Objectives:**

The study aims to assess the socioeconomic inequalities on unmet needs for mental health care in EU countries in 2019.

**Methods:**

This was a cross-sectional study using data from the 2019 European Health Interview Survey across 26 EU countries. The main outcome considered was the proportion of self-reported unmet needs for mental health care due to financial reasons. Inequalities for income, education and degree of urbanization were assessed by calculating the rural-city, primary-tertiary education and lowest-highest income quintiles, respectively.

**Results:**

The proportion of self-reported unmet need for mental health care in 2019 ranged between 1.1% (Romania) and 27.8% (Portugal), with a median of 3.6%. Regarding income inequality, all countries except Hungary (ratio=0.88) showed highest share of unmet need among inhabitants with the lowest income quintile. The country with the highest inequality was Greece with a ratio of 23.8. Regarding education inequality, 15 out of 26 countries showed that less educated inhabitants had highest unmet needs of mental health care, with values ranging from 0.5 in the Netherlands and 7.2 in Bulgaria. As for degree of urbanization, rurality showed lowest unmet needs for 21 out of 26 countries, with the highest ratio being 2 in Romania.

**Conclusions:**

The study highlights significant and wide socioeconomic (income, education, and urbanization) inequalities in unmet mental health care needs across EU countries.

While income inequality plays a similar role across EU countries with the poorer quintile showing higher unmet needs due to financial reasons, EU is divided on the role that education plays. On the opposite side, there is also a tendency across the EU for rural areas showing lower unmet needs for mental health care. Policymakers should prioritize strategies to ensure financial access to mental health services, as well as promoting mental health literacy and improve service availability for vulnerable populations.

**Disclosure of Interest:**

J. V. Santos Conflict with: This article was supported by National Funds through FCT - Fundação para a Ciência e a Tecnologia, I.P., within CINTESIS, R&D Unit (reference UIDB/4255/2020), V. Pinheiro: None Declared

